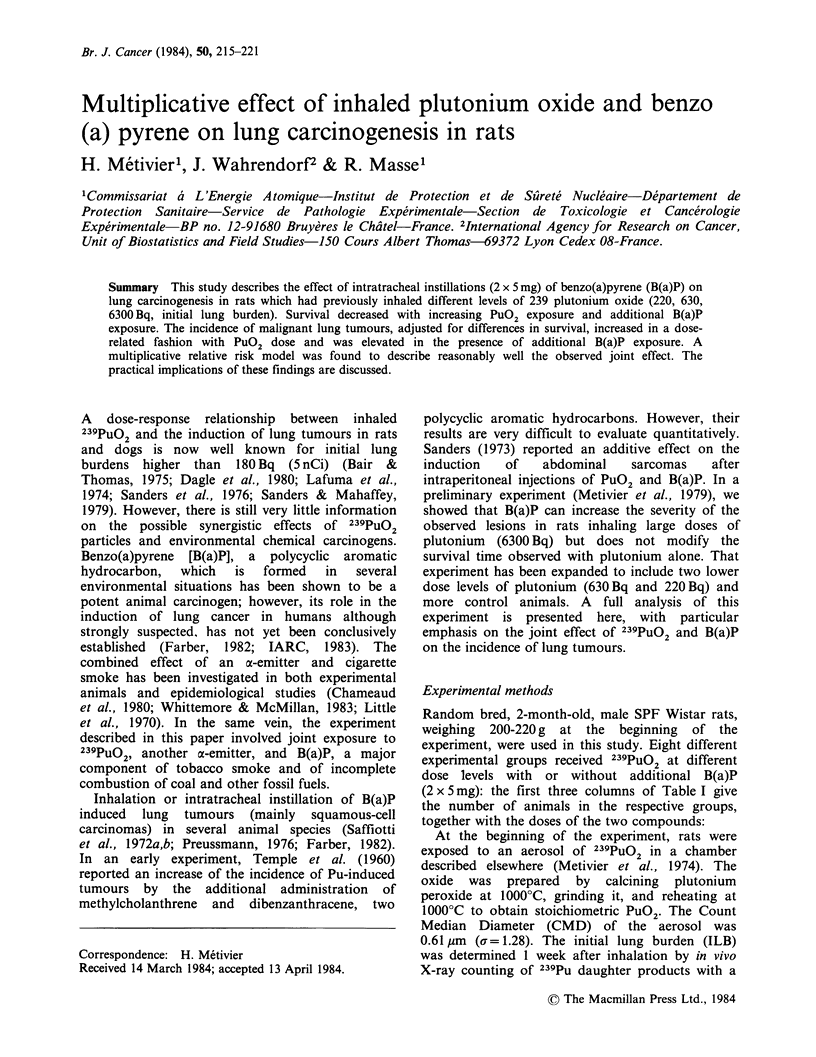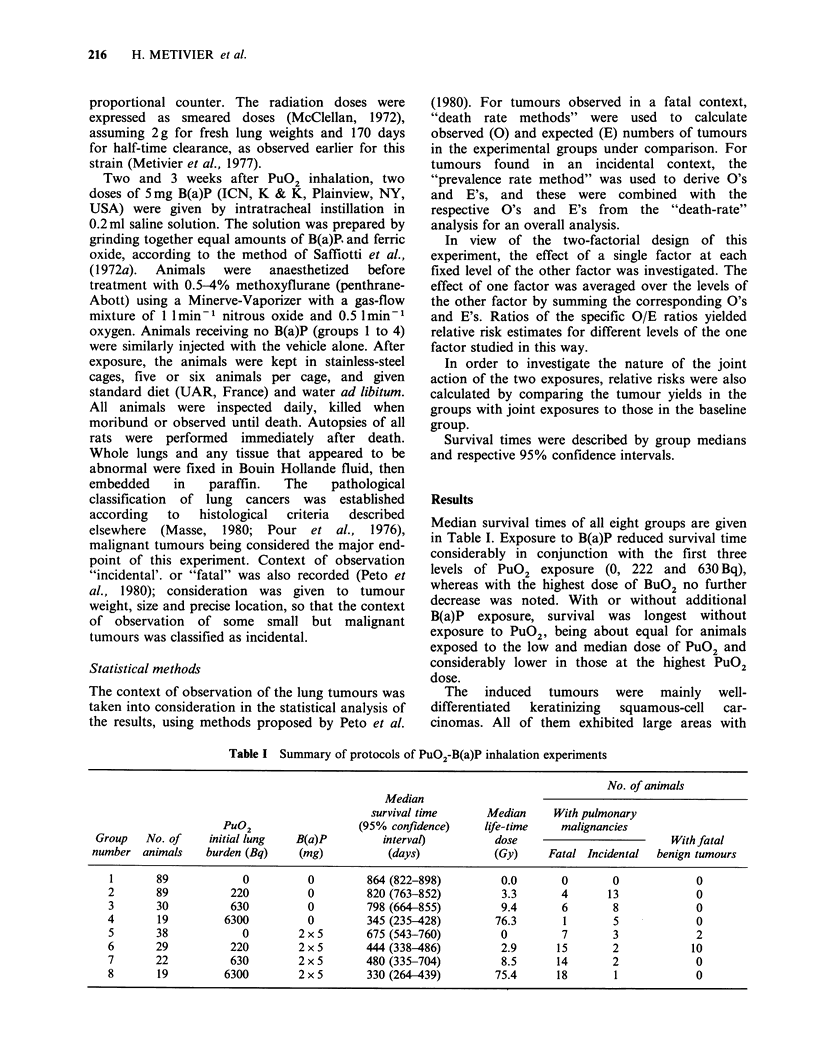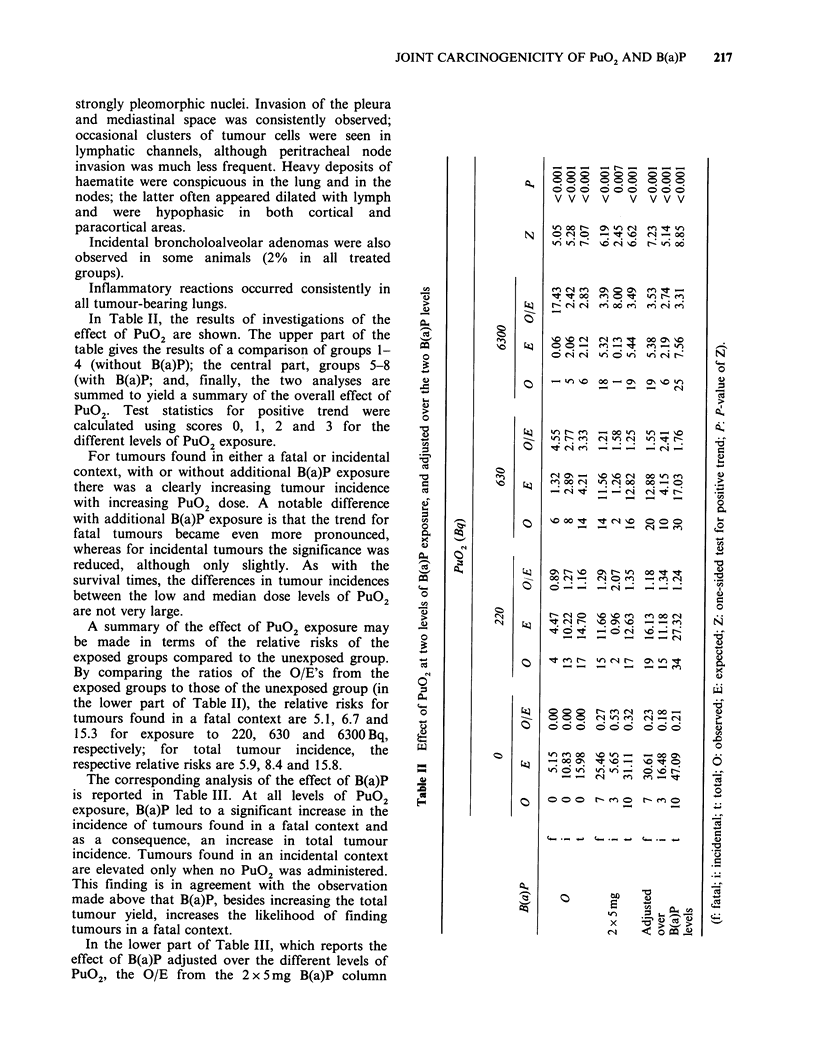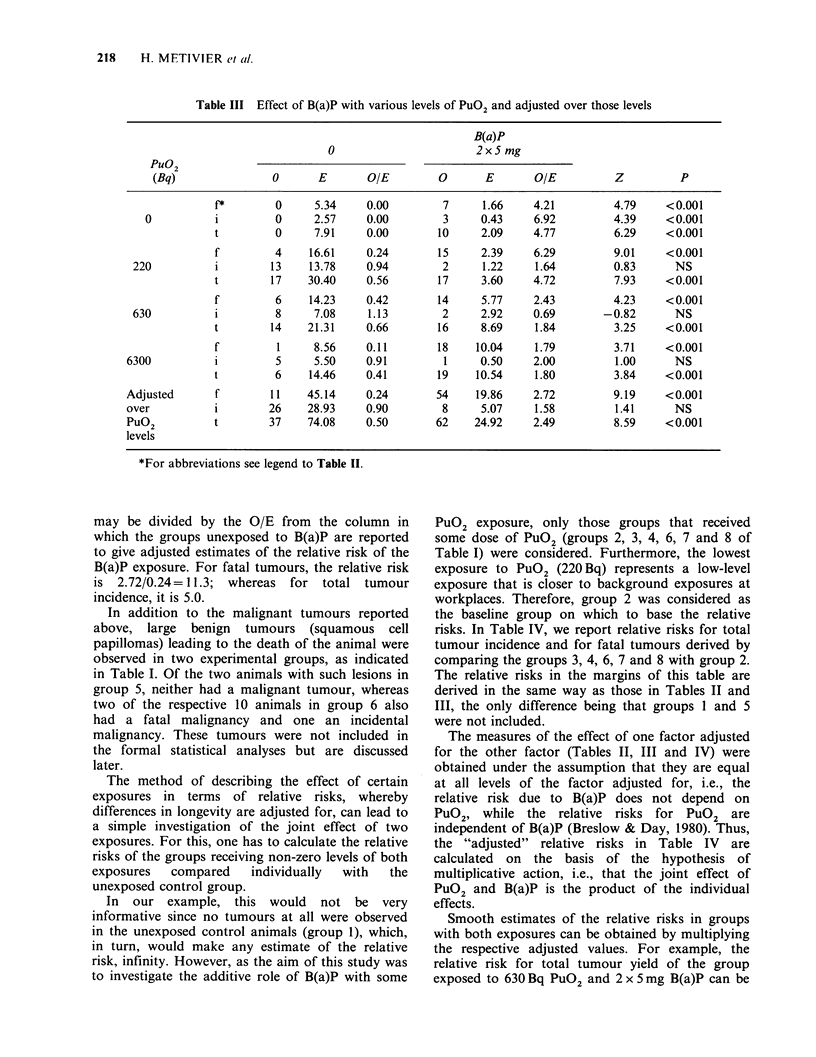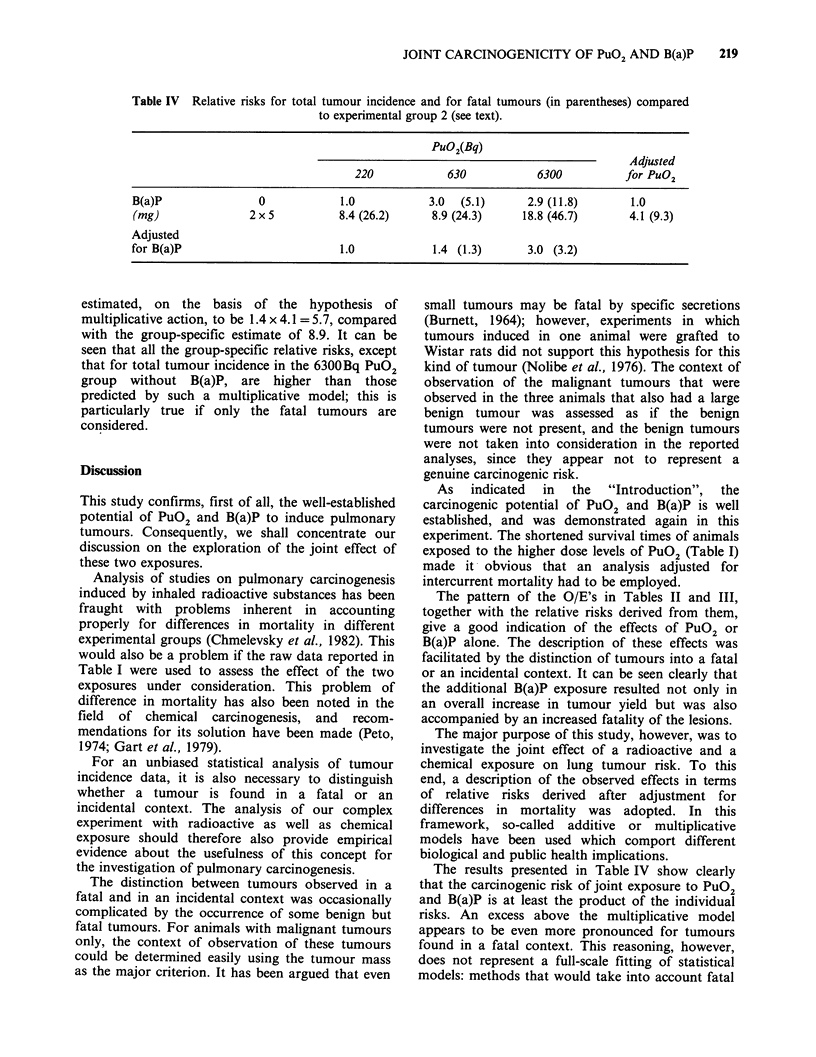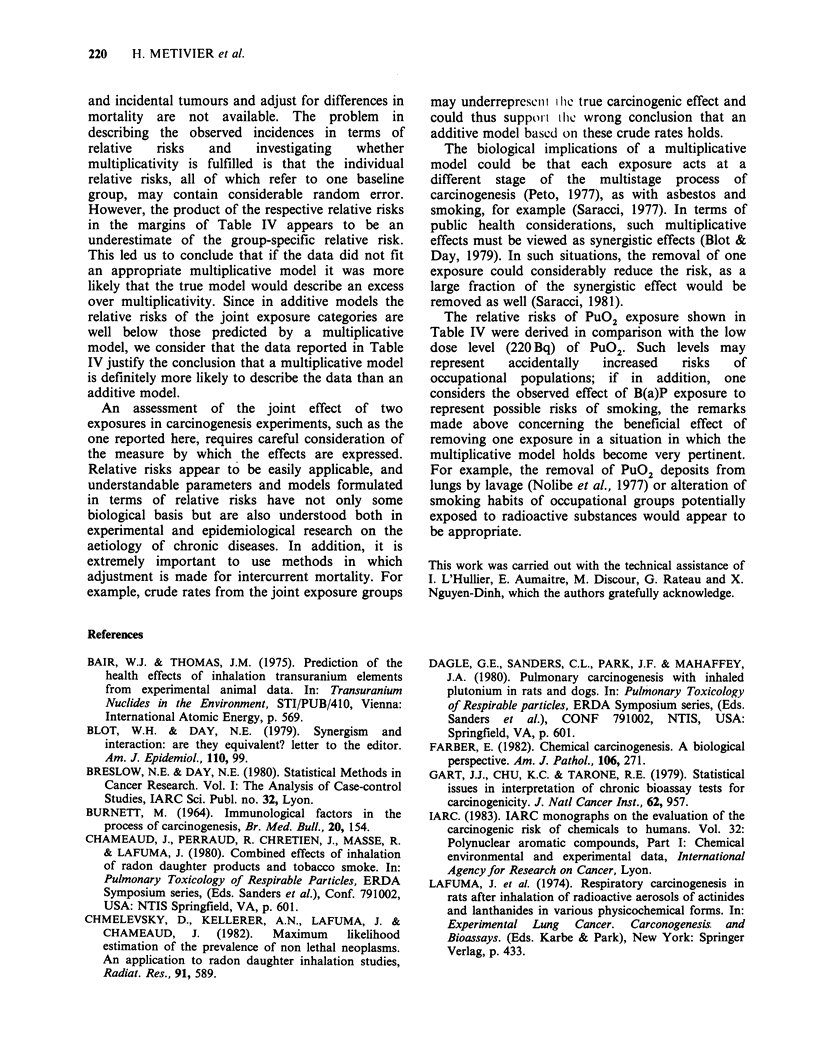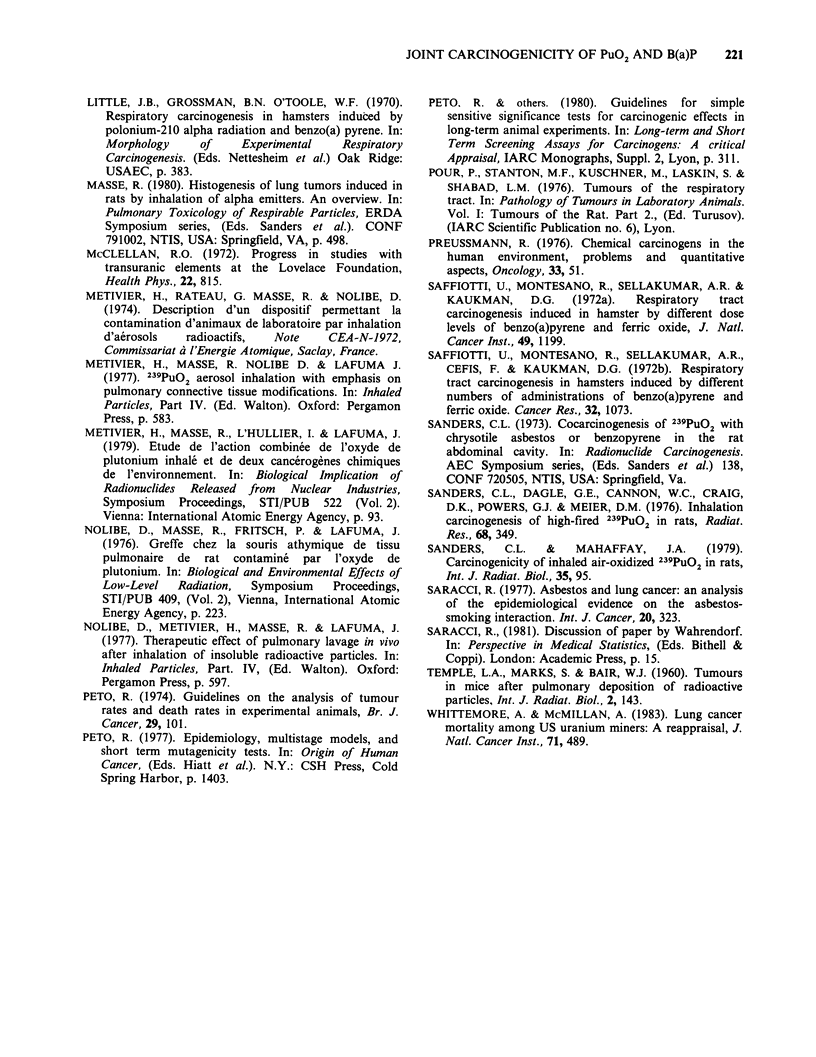# Multiplicative effect of inhaled plutonium oxide and benzo (a) pyrene on lung carcinogenesis in rats.

**DOI:** 10.1038/bjc.1984.165

**Published:** 1984-08

**Authors:** H. Métivier, J. Wahrendorf, R. Masse

## Abstract

This study describes the effect of intratracheal instillations (2 X 5 mg) of benzo(a)pyrene (B(a)P) on lung carcinogenesis in rats which had previously inhaled different levels of 239 plutonium oxide (220, 630, 6300 Bq, initial lung burden). Survival decreased with increasing PuO2 exposure and additional B(a)P exposure. The incidence of malignant lung tumours, adjusted for differences in survival, increased in a dose-related fashion with PuO2 dose and was elevated in the presence of additional B(a)P exposure. A multiplicative relative risk model was found to describe reasonably well the observed joint effect. The practical implications of these findings are discussed.


*Br. J. Cancer* (1984), **50**, 215–221

### Multiplicative effect of inhaled plutonium oxide and benzo (a) pyrene on lung carcinogenesis in rats

H. Métivier[1], J. Wahrendorf[2] & R. Masse[1]

[1]*Commissariat á L'Energie Atomique—Institut de Protection et de Sûreté Nucléaire—Département de Protection Sanitaire—Service de Pathologie Expérimentale—Section de Toxicologie et Cancérologie Expérimentale—BP no. 12-91680 Bruyères le Châtel—France.* [2]*International Agency for Research on Cancer, Unit of Biostatistics and Field Studies—150 Cours Albert Thomas—69372 Lyon Cedex 08-France.*

**Summary** This study describes the effect of intratracheal instillations (2 × 5 mg) of benzo(a)pyrene (B(a)P) on lung carcinogenesis in rats which had previously inhaled different levels of 239 plutonium oxide (220, 630, 6300 Bq, initial lung burden). Survival decreased with increasing $PuO_2$ exposure and additional B(a)P exposure. The incidence of malignant lung tumours, adjusted for differences in survival, increased in a dose-related fashion with $PuO_2$ dose and was elevated in the presence of additional B(a)P exposure. A multiplicative relative risk model was found to describe reasonably well the observed joint effect. The practical implications of these findings are discussed.

A dose-response relationship between inhaled $^{239}PuO_2$ and the induction of lung tumours in rats and dogs is now well known for initial lung burdens higher than 180 Bq (5 nCi) (Bair & Thomas, 1975; Dagle *et al.*, 1980; Lafuma *et al.*, 1974; Sanders *et al.*, 1976; Sanders & Mahaffey, 1979). However, there is still very little information on the possible synergistic effects of $^{239}PuO_2$ particles and environmental chemical carcinogens. Benzo(a)pyrene [B(a)P], a polycyclic aromatic hydrocarbon, which is formed in several environmental situations has been shown to be a potent animal carcinogen; however, its role in the induction of lung cancer in humans although strongly suspected, has not yet been conclusively established (Farber, 1982; IARC, 1983). The combined effect of an α-emitter and cigarette smoke has been investigated in both experimental animals and epidemiological studies (Chameaud *et al.*, 1980; Whittemore & McMillan, 1983; Little *et al.*, 1970). In the same vein, the experiment described in this paper involved joint exposure to $^{239}PuO_2$, another α-emitter, and B(a)P, a major component of tobacco smoke and of incomplete combustion of coal and other fossil fuels.

Inhalation or intratracheal instillation of B(a)P induced lung tumours (mainly squamous-cell carcinomas) in several animal species (Saffiotti *et al.*, 1972*a,b*; Preussmann, 1976; Farber, 1982). In an early experiment, Temple *et al.* (1960) reported an increase of the incidence of Pu-induced tumours by the additional administration of methylcholanthrene and dibenzanthracene, two polycyclic aromatic hydrocarbons. However, their results are very difficult to evaluate quantitatively. Sanders (1973) reported an additive effect on the induction of abdominal sarcomas after intraperitoneal injections of $PuO_2$ and B(a)P. In a preliminary experiment (Metivier *et al.*, 1979), we showed that B(a)P can increase the severity of the observed lesions in rats inhaling large doses of plutonium (6300 Bq) but does not modify the survival time observed with plutonium alone. That experiment has been expanded to include two lower dose levels of plutonium (630 Bq and 220 Bq) and more control animals. A full analysis of this experiment is presented here, with particular emphasis on the joint effect of $^{239}PuO_2$ and B(a)P on the incidence of lung tumours.

### *Experimental methods*

Random bred, 2-month-old, male SPF Wistar rats, weighing 200-220 g at the beginning of the experiment, were used in this study. Eight different experimental groups received $^{239}PuO_2$ at different dose levels with or without additional B(a)P (2 × 5 mg): the first three columns of Table I give the number of animals in the respective groups, together with the doses of the two compounds:

At the beginning of the experiment, rats were exposed to an aerosol of $^{239}PuO_2$ in a chamber described elsewhere (Metivier *et al.*, 1974). The oxide was prepared by calcining plutonium peroxide at 1000°C, grinding it, and reheating at 1000°C to obtain stoichiometric $PuO_2$. The Count Median Diameter (CMD) of the aerosol was 0.61 μm ($\sigma = 1.28$). The initial lung burden (ILB) was determined 1 week after inhalation by *in vivo* X-ray counting of $^{239}Pu$ daughter products with a

Correspondence: H. Métivier
Received 14 March 1984; accepted 13 April 1984.

© The Macmillan Press Ltd., 1984

proportional counter. The radiation doses were expressed as smeared doses (McClellan, 1972), assuming 2 g for fresh lung weights and 170 days for half-time clearance, as observed earlier for this strain (Metivier *et al.*, 1977).

Two and 3 weeks after $PuO_2$ inhalation, two doses of 5 mg B(a)P (ICN, K & K, Plainview, NY, USA) were given by intratracheal instillation in 0.2 ml saline solution. The solution was prepared by grinding together equal amounts of B(a)P and ferric oxide, according to the method of Saffiotti *et al.*, (1972*a*). Animals were anaesthetized before treatment with 0.5–4% methoxyflurane (penthrane-Abott) using a Minerve-Vaporizer with a gas-flow mixture of 1 l $min^{-1}$ nitrous oxide and 0.5 l $min^{-1}$ oxygen. Animals receiving no B(a)P (groups 1 to 4) were similarly injected with the vehicle alone. After exposure, the animals were kept in stainless-steel cages, five or six animals per cage, and given standard diet (UAR, France) and water *ad libitum*. All animals were inspected daily, killed when moribund or observed until death. Autopsies of all rats were performed immediately after death. Whole lungs and any tissue that appeared to be abnormal were fixed in Bouin Hollande fluid, then embedded in paraffin. The pathological classification of lung cancers was established according to histological criteria described elsewhere (Masse, 1980; Pour *et al.*, 1976), malignant tumours being considered the major end-point of this experiment. Context of observation "incidental'. or "fatal" was also recorded (Peto *et al.*, 1980); consideration was given to tumour weight, size and precise location, so that the context of observation of some small but malignant tumours was classified as incidental.

*Statistical methods*

The context of observation of the lung tumours was taken into consideration in the statistical analysis of the results, using methods proposed by Peto *et al.* (1980). For tumours observed in a fatal context, "death rate methods" were used to calculate observed (O) and expected (E) numbers of tumours in the experimental groups under comparison. For tumours found in an incidental context, the "prevalence rate method" was used to derive O's and E's, and these were combined with the respective O's and E's from the "death-rate" analysis for an overall analysis.

In view of the two-factorial design of this experiment, the effect of a single factor at each fixed level of the other factor was investigated. The effect of one factor was averaged over the levels of the other factor by summing the corresponding O's and E's. Ratios of the specific O/E ratios yielded relative risk estimates for different levels of the one factor studied in this way.

In order to investigate the nature of the joint action of the two exposures, relative risks were also calculated by comparing the tumour yields in the groups with joint exposures to those in the baseline group.

Survival times were described by group medians and respective 95% confidence intervals.

### Results

Median survival times of all eight groups are given in Table I. Exposure to B(a)P reduced survival time considerably in conjunction with the first three levels of $PuO_2$ exposure (0, 222 and 630 Bq), whereas with the highest dose of $BuO_2$ no further decrease was noted. With or without additional B(a)P exposure, survival was longest without exposure to $PuO_2$, being about equal for animals exposed to the low and median dose of $PuO_2$ and considerably lower in those at the highest $PuO_2$ dose.

The induced tumours were mainly well-differentiated keratinizing squamous-cell carcinomas. All of them exhibited large areas with

**Table I** Summary of protocols of $PuO_2$-B(a)P inhalation experiments

| Group number | No. of animals | $PuO_2$ initial lung burden (Bq) | B(a)P (mg) | Median survival time (95% confidence) interval) (days) | Median life-time dose (Gy) | No. of animals: With pulmonary malignancies: Fatal | No. of animals: With pulmonary malignancies: Incidental | No. of animals: With fatal benign tumours |
|---|---|---|---|---|---|---|---|---|
| 1 | 89 | 0 | 0 | 864 (822–898) | 0.0 | 0 | 0 | 0 |
| 2 | 89 | 220 | 0 | 820 (763–852) | 3.3 | 4 | 13 | 0 |
| 3 | 30 | 630 | 0 | 798 (664–855) | 9.4 | 6 | 8 | 0 |
| 4 | 19 | 6300 | 0 | 345 (235–428) | 76.3 | 1 | 5 | 0 |
| 5 | 38 | 0 | 2×5 | 675 (543–760) | 0 | 7 | 3 | 2 |
| 6 | 29 | 220 | 2×5 | 444 (338–486) | 2.9 | 15 | 2 | 10 |
| 7 | 22 | 630 | 2×5 | 480 (335–704) | 8.5 | 14 | 2 | 0 |
| 8 | 19 | 6300 | 2×5 | 330 (264–439) | 75.4 | 18 | 1 | 0 |

strongly pleomorphic nuclei. Invasion of the pleura and mediastinal space was consistently observed; occasional clusters of tumour cells were seen in lymphatic channels, although peritracheal node invasion was much less frequent. Heavy deposits of haematite were conspicuous in the lung and in the nodes; the latter often appeared dilated with lymph and were hypophasic in both cortical and paracortical areas.

Incidental broncholoalveolar adenomas were also observed in some animals (2% in all treated groups).

Inflammatory reactions occurred consistently in all tumour-bearing lungs.

In Table II, the results of investigations of the effect of $PuO_2$ are shown. The upper part of the table gives the results of a comparison of groups 1–4 (without B(a)P); the central part, groups 5–8 (with B(a)P; and, finally, the two analyses are summed to yield a summary of the overall effect of $PuO_2$. Test statistics for positive trend were calculated using scores 0, 1, 2 and 3 for the different levels of $PuO_2$ exposure.

For tumours found in either a fatal or incidental context, with or without additional B(a)P exposure there was a clearly increasing tumour incidence with increasing $PuO_2$ dose. A notable difference with additional B(a)P exposure is that the trend for fatal tumours became even more pronounced, whereas for incidental tumours the significance was reduced, although only slightly. As with the survival times, the differences in tumour incidences between the low and median dose levels of $PuO_2$ are not very large.

A summary of the effect of $PuO_2$ exposure may be made in terms of the relative risks of the exposed groups compared to the unexposed group. By comparing the ratios of the O/E's from the exposed groups to those of the unexposed group (in the lower part of Table II), the relative risks for tumours found in a fatal context are 5.1, 6.7 and 15.3 for exposure to 220, 630 and 6300 Bq, respectively; for total tumour incidence, the respective relative risks are 5.9, 8.4 and 15.8.

The corresponding analysis of the effect of B(a)P is reported in Table III. At all levels of $PuO_2$ exposure, B(a)P led to a significant increase in the incidence of tumours found in a fatal context and as a consequence, an increase in total tumour incidence. Tumours found in an incidental context are elevated only when no $PuO_2$ was administered. This finding is in agreement with the observation made above that B(a)P, besides increasing the total tumour yield, increases the likelihood of finding tumours in a fatal context.

In the lower part of Table III, which reports the effect of B(a)P adjusted over the different levels of $PuO_2$, the O/E from the 2 × 5 mg B(a)P column

**Table II** Effect of $PuO_2$ at two levels of B(a)P exposure, and adjusted over the two B(a)P levels

| | | $PuO_2$ (Bq) | | | | | | | | | | | | | |
|---|---|---|---|---|---|---|---|---|---|---|---|---|---|---|---|
| | | 0 | | | 220 | | | 630 | | | 6300 | | | | |
| *B(a)P* | | *O* | *E* | *O/E* | *O* | *E* | *O/E* | *O* | *E* | *O/E* | *O* | *E* | *O/E* | *Z* | *P* |
| 0 | f | 0 | 5.15 | 0.00 | 4 | 4.47 | 0.89 | 6 | 1.32 | 4.55 | 1 | 0.06 | 17.43 | 5.05 | <0.001 |
| | i | 0 | 10.83 | 0.00 | 13 | 10.22 | 1.27 | 8 | 2.89 | 2.77 | 5 | 2.06 | 2.42 | 5.28 | <0.001 |
| | t | 0 | 15.98 | 0.00 | 17 | 14.70 | 1.16 | 14 | 4.21 | 3.33 | 6 | 2.12 | 2.83 | 7.07 | <0.001 |
| 2 × 5 mg | f | 7 | 25.46 | 0.27 | 15 | 11.66 | 1.29 | 14 | 11.56 | 1.21 | 18 | 5.32 | 3.39 | 6.19 | <0.001 |
| | i | 3 | 5.65 | 0.53 | 2 | 0.96 | 2.07 | 2 | 1.26 | 1.58 | 1 | 0.13 | 8.00 | 2.45 | 0.007 |
| | t | 10 | 31.11 | 0.32 | 17 | 12.63 | 1.35 | 16 | 12.82 | 1.25 | 19 | 5.44 | 3.49 | 6.62 | <0.001 |
| Adjusted over B(a)P levels | f | 7 | 30.61 | 0.23 | 19 | 16.13 | 1.18 | 20 | 12.88 | 1.55 | 19 | 5.38 | 3.53 | 7.23 | <0.001 |
| | i | 3 | 16.48 | 0.18 | 15 | 11.18 | 1.34 | 10 | 4.15 | 2.41 | 6 | 2.19 | 2.74 | 5.14 | <0.001 |
| | t | 10 | 47.09 | 0.21 | 34 | 27.32 | 1.24 | 30 | 17.03 | 1.76 | 25 | 7.56 | 3.31 | 8.85 | <0.001 |

(f: fatal; i: incidental; t: total; O: observed; E: expected; Z: one-sided test for positive trend; P: P-value of Z).

**Table III** Effect of B(a)P with various levels of $PuO_2$ and adjusted over those levels

| *$PuO_2$ (Bq)* | | *B(a)P 0* | | | *B(a)P 2×5 mg* | | | | |
|---|---|---|---|---|---|---|---|---|---|
| | | *O* | *E* | *O/E* | *O* | *E* | *O/E* | *Z* | *P* |
| 0 | f* | 0 | 5.34 | 0.00 | 7 | 1.66 | 4.21 | 4.79 | <0.001 |
| | i | 0 | 2.57 | 0.00 | 3 | 0.43 | 6.92 | 4.39 | <0.001 |
| | t | 0 | 7.91 | 0.00 | 10 | 2.09 | 4.77 | 6.29 | <0.001 |
| 220 | f | 4 | 16.61 | 0.24 | 15 | 2.39 | 6.29 | 9.01 | <0.001 |
| | i | 13 | 13.78 | 0.94 | 2 | 1.22 | 1.64 | 0.83 | NS |
| | t | 17 | 30.40 | 0.56 | 17 | 3.60 | 4.72 | 7.93 | <0.001 |
| 630 | f | 6 | 14.23 | 0.42 | 14 | 5.77 | 2.43 | 4.23 | <0.001 |
| | i | 8 | 7.08 | 1.13 | 2 | 2.92 | 0.69 | −0.82 | NS |
| | t | 14 | 21.31 | 0.66 | 16 | 8.69 | 1.84 | 3.25 | <0.001 |
| 6300 | f | 1 | 8.56 | 0.11 | 18 | 10.04 | 1.79 | 3.71 | <0.001 |
| | i | 5 | 5.50 | 0.91 | 1 | 0.50 | 2.00 | 1.00 | NS |
| | t | 6 | 14.46 | 0.41 | 19 | 10.54 | 1.80 | 3.84 | <0.001 |
| Adjusted over $PuO_2$ levels | f | 11 | 45.14 | 0.24 | 54 | 19.86 | 2.72 | 9.19 | <0.001 |
| | i | 26 | 28.93 | 0.90 | 8 | 5.07 | 1.58 | 1.41 | NS |
| | t | 37 | 74.08 | 0.50 | 62 | 24.92 | 2.49 | 8.59 | <0.001 |

*For abbreviations see legend to **Table II**.

may be divided by the O/E from the column in which the groups unexposed to B(a)P are reported to give adjusted estimates of the relative risk of the B(a)P exposure. For fatal tumours, the relative risk is 2.72/0.24 = 11.3; whereas for total tumour incidence, it is 5.0.

In addition to the malignant tumours reported above, large benign tumours (squamous cell papillomas) leading to the death of the animal were observed in two experimental groups, as indicated in Table I. Of the two animals with such lesions in group 5, neither had a malignant tumour, whereas two of the respective 10 animals in group 6 also had a fatal malignancy and one an incidental malignancy. These tumours were not included in the formal statistical analyses but are discussed later.

The method of describing the effect of certain exposures in terms of relative risks, whereby differences in longevity are adjusted for, can lead to a simple investigation of the joint effect of two exposures. For this, one has to calculate the relative risks of the groups receiving non-zero levels of both exposures compared individually with the unexposed control group.

In our example, this would not be very informative since no tumours at all were observed in the unexposed control animals (group 1), which, in turn, would make any estimate of the relative risk, infinity. However, as the aim of this study was to investigate the additive role of B(a)P with some $PuO_2$ exposure, only those groups that received some dose of $PuO_2$ (groups 2, 3, 4, 6, 7 and 8 of Table I) were considered. Furthermore, the lowest exposure to $PuO_2$ (220 Bq) represents a low-level exposure that is closer to background exposures at workplaces. Therefore, group 2 was considered as the baseline group on which to base the relative risks. In Table IV, we report relative risks for total tumour incidence and for fatal tumours derived by comparing the groups 3, 4, 6, 7 and 8 with group 2. The relative risks in the margins of this table are derived in the same way as those in Tables II and III, the only difference being that groups 1 and 5 were not included.

The measures of the effect of one factor adjusted for the other factor (Tables II, III and IV) were obtained under the assumption that they are equal at all levels of the factor adjusted for, i.e., the relative risk due to B(a)P does not depend on $PuO_2$, while the relative risks for $PuO_2$ are independent of B(a)P (Breslow & Day, 1980). Thus, the "adjusted" relative risks in Table IV are calculated on the basis of the hypothesis of multiplicative action, i.e., that the joint effect of $PuO_2$ and B(a)P is the product of the individual effects.

Smooth estimates of the relative risks in groups with both exposures can be obtained by multiplying the respective adjusted values. For example, the relative risk for total tumour yield of the group exposed to 630 Bq $PuO_2$ and 2 × 5 mg B(a)P can be

**Table IV** Relative risks for total tumour incidence and for fatal tumours (in parentheses) compared to experimental group 2 (see text).

| | | *$PuO_2$(Bq)* | | | |
|---|---|---|---|---|---|
| | | *220* | *630* | *6300* | *Adjusted for $PuO_2$* |
| B(a)P | 0 | 1.0 | 3.0 (5.1) | 2.9 (11.8) | 1.0 |
| *(mg)* | 2×5 | 8.4 (26.2) | 8.9 (24.3) | 18.8 (46.7) | 4.1 (9.3) |
| Adjusted for B(a)P | | 1.0 | 1.4 (1.3) | 3.0 (3.2) | |

estimated, on the basis of the hypothesis of multiplicative action, to be $1.4 \times 4.1 = 5.7$, compared with the group-specific estimate of 8.9. It can be seen that all the group-specific relative risks, except that for total tumour incidence in the 6300 Bq $PuO_2$ group without B(a)P, are higher than those predicted by such a multiplicative model; this is particularly true if only the fatal tumours are considered.

### Discussion

This study confirms, first of all, the well-established potential of $PuO_2$ and B(a)P to induce pulmonary tumours. Consequently, we shall concentrate our discussion on the exploration of the joint effect of these two exposures.

Analysis of studies on pulmonary carcinogenesis induced by inhaled radioactive substances has been fraught with problems inherent in accounting properly for differences in mortality in different experimental groups (Chmelevsky *et al.*, 1982). This would also be a problem if the raw data reported in Table I were used to assess the effect of the two exposures under consideration. This problem of difference in mortality has also been noted in the field of chemical carcinogenesis, and recommendations for its solution have been made (Peto, 1974; Gart *et al.*, 1979).

For an unbiased statistical analysis of tumour incidence data, it is also necessary to distinguish whether a tumour is found in a fatal or an incidental context. The analysis of our complex experiment with radioactive as well as chemical exposure should therefore also provide empirical evidence about the usefulness of this concept for the investigation of pulmonary carcinogenesis.

The distinction between tumours observed in a fatal and in an incidental context was occasionally complicated by the occurrence of some benign but fatal tumours. For animals with malignant tumours only, the context of observation of these tumours could be determined easily using the tumour mass as the major criterion. It has been argued that even small tumours may be fatal by specific secretions (Burnett, 1964); however, experiments in which tumours induced in one animal were grafted to Wistar rats did not support this hypothesis for this kind of tumour (Nolibe *et al.*, 1976). The context of observation of the malignant tumours that were observed in the three animals that also had a large benign tumour was assessed as if the benign tumours were not present, and the benign tumours were not taken into consideration in the reported analyses, since they appear not to represent a genuine carcinogenic risk.

As indicated in the "Introduction", the carcinogenic potential of $PuO_2$ and B(a)P is well established, and was demonstrated again in this experiment. The shortened survival times of animals exposed to the higher dose levels of $PuO_2$ (Table I) made it obvious that an analysis adjusted for intercurrent mortality had to be employed.

The pattern of the O/E's in Tables II and III, together with the relative risks derived from them, give a good indication of the effects of $PuO_2$ or B(a)P alone. The description of these effects was facilitated by the distinction of tumours into a fatal or an incidental context. It can be seen clearly that the additional B(a)P exposure resulted not only in an overall increase in tumour yield but was also accompanied by an increased fatality of the lesions.

The major purpose of this study, however, was to investigate the joint effect of a radioactive and a chemical exposure on lung tumour risk. To this end, a description of the observed effects in terms of relative risks derived after adjustment for differences in mortality was adopted. In this framework, so-called additive or multiplicative models have been used which comport different biological and public health implications.

The results presented in Table IV show clearly that the carcinogenic risk of joint exposure to $PuO_2$ and B(a)P is at least the product of the individual risks. An excess above the multiplicative model appears to be even more pronounced for tumours found in a fatal context. This reasoning, however, does not represent a full-scale fitting of statistical models: methods that would take into account fatal and incidental tumours and adjust for differences in mortality are not available. The problem in describing the observed incidences in terms of relative risks and investigating whether multiplicativity is fulfilled is that the individual relative risks, all of which refer to one baseline group, may contain considerable random error. However, the product of the respective relative risks in the margins of Table IV appears to be an underestimate of the group-specific relative risk. This led us to conclude that if the data did not fit an appropriate multiplicative model it was more likely that the true model would describe an excess over multiplicativity. Since in additive models the relative risks of the joint exposure categories are well below those predicted by a multiplicative model, we consider that the data reported in Table IV justify the conclusion that a multiplicative model is definitely more likely to describe the data than an additive model.

An assessment of the joint effect of two exposures in carcinogenesis experiments, such as the one reported here, requires careful consideration of the measure by which the effects are expressed. Relative risks appear to be easily applicable, and understandable parameters and models formulated in terms of relative risks have not only some biological basis but are also understood both in experimental and epidemiological research on the aetiology of chronic diseases. In addition, it is extremely important to use methods in which adjustment is made for intercurrent mortality. For example, crude rates from the joint exposure groups may underrepresent the true carcinogenic effect and could thus support the wrong conclusion that an additive model based on these crude rates holds.

The biological implications of a multiplicative model could be that each exposure acts at a different stage of the multistage process of carcinogenesis (Peto, 1977), as with asbestos and smoking, for example (Saracci, 1977). In terms of public health considerations, such multiplicative effects must be viewed as synergistic effects (Blot & Day, 1979). In such situations, the removal of one exposure could considerably reduce the risk, as a large fraction of the synergistic effect would be removed as well (Saracci, 1981).

The relative risks of $PuO_2$ exposure shown in Table IV were derived in comparison with the low dose level (220 Bq) of $PuO_2$. Such levels may represent accidentally increased risks of occupational populations; if in addition, one considers the observed effect of B(a)P exposure to represent possible risks of smoking, the remarks made above concerning the beneficial effect of removing one exposure in a situation in which the multiplicative model holds become very pertinent. For example, the removal of $PuO_2$ deposits from lungs by lavage (Nolibe *et al.*, 1977) or alteration of smoking habits of occupational groups potentially exposed to radioactive substances would appear to be appropriate.

This work was carried out with the technical assistance of I. L'Hullier, E. Aumaitre, M. Discour, G. Rateau and X. Nguyen-Dinh, which the authors gratefully acknowledge.

### References

BAIR, W.J. & THOMAS, J.M. (1975). Prediction of the health effects of inhalation transuranium elements from experimental animal data. In: *Transuranium Nuclides in the Environment*, STI/PUB/410, Vienna: International Atomic Energy, p. 569.

BLOT, W.H. & DAY, N.E. (1979). Synergism and interaction: are they equivalent? letter to the editor. *Am. J. Epidemiol.*, **110**, 99.

BRESLOW, N.E. & DAY, N.E. (1980). Statistical Methods in Cancer Research. Vol. I: The Analysis of Case-control Studies, IARC Sci. Publ. no. **32**, Lyon.

BURNETT, M. (1964). Immunological factors in the process of carcinogenesis, *Br. Med. Bull.*, **20**, 154.

CHAMEAUD, J., PERRAUD, R. CHRETIEN, J., MASSE, R. & LAFUMA, J. (1980). Combined effects of inhalation of radon daughter products and tobacco smoke. In: *Pulmonary Toxicology of Respirable Particles*, ERDA Symposium series, (Eds. Sanders *et al.*), Conf. 791002, USA: NTIS Springfield, VA, p. 601.

CHMELEVSKY, D., KELLERER, A.N., LAFUMA, J. & CHAMEAUD, J. (1982). Maximum likelihood estimation of the prevalence of non lethal neoplasms. An application to radon daughter inhalation studies, *Radiat. Res.*, **91**, 589.

DAGLE, G.E., SANDERS, C.L., PARK, J.F. & MAHAFFEY, J.A. (1980). Pulmonary carcinogenesis with inhaled plutonium in rats and dogs. In: *Pulmonary Toxicology of Respirable particles*, ERDA Symposium series, (Eds. Sanders *et al.*), CONF 791002, NTIS, USA: Springfield, VA, p. 601.

FARBER, E. (1982). Chemical carcinogenesis. A biological perspective. *Am. J. Pathol.*, **106**, 271.

GART, J.J., CHU, K.C. & TARONE, R.E. (1979). Statistical issues in interpretation of chronic bioassay tests for carcinogenicity. *J. Natl Cancer Inst.*, **62**, 957.

IARC. (1983). IARC monographs on the evaluation of the carcinogenic risk of chemicals to humans. Vol. 32: Polynuclear aromatic compounds, Part I: Chemical environmental and experimental data, *International Agency for Research on Cancer*, Lyon.

LAFUMA, J. *et al.* (1974). Respiratory carcinogenesis in rats after inhalation of radioactive aerosols of actinides and lanthanides in various physicochemical forms. In: *Experimental Lung Cancer. Carconogenesis and Bioassays*. (Eds. Karbe & Park), New York: Springer Verlag, p. 433.

LITTLE, J.B., GROSSMAN, B.N. O'TOOLE, W.F. (1970). Respiratory carcinogenesis in hamsters induced by polonium-210 alpha radiation and benzo(a) pyrene. In: *Morphology of Experimental Respiratory Carcinogenesis*. (Eds. Nettesheim *et al.*) Oak Ridge: USAEC, p. 383.

MASSE, R. (1980). Histogenesis of lung tumors induced in rats by inhalation of alpha emitters. An overview. In: *Pulmonary Toxicology of Respirable Particles*, ERDA Symposium series, (Eds. Sanders *et al.*). CONF 791002, NTIS, USA: Springfield, VA, p. 498.

McCLELLAN, R.O. (1972). Progress in studies with transuranic elements at the Lovelace Foundation, *Health Phys.*, **22**, 815.

METIVIER, H., RATEAU, G. MASSE, R. & NOLIBE, D. (1974). Description d'un dispositif permettant la contamination d'animaux de laboratoire par inhalation d'aérosols radioactifs, *Note CEA-N-1972, Commissariat à l'Energie Atomique, Saclay, France*.

METIVIER, H., MASSE, R. NOLIBE D. & LAFUMA J. (1977). $^{239}PuO_2$ aerosol inhalation with emphasis on pulmonary connective tissue modifications. In: *Inhaled Particles*, Part IV. (Ed. Walton). Oxford: Pergamon Press, p. 583.

METIVIER, H., MASSE, R., L'HULLIER, I. & LAFUMA, J. (1979). Etude de l'action combinée de l'oxyde de plutonium inhalé et de deux cancérogènes chimiques de l'environnement. In: *Biological Implication of Radionuclides Released from Nuclear Industries*, Symposium Proceedings, STI/PUB 522 (Vol. 2). Vienna: International Atomic Energy Agency, p. 93.

NOLIBE, D., MASSE, R., FRITSCH, P. & LAFUMA, J. (1976). Greffe chez la souris athymique de tissu pulmonaire de rat contaminé par l'oxyde de plutonium. In: *Biological and Environmental Effects of Low-Level Radiation*, Symposium Proceedings, STI/PUB 409, (Vol. 2), Vienna, International Atomic Energy Agency, p. 223.

NOLIBE, D., METIVIER, H., MASSE, R. & LAFUMA, J. (1977). Therapeutic effect of pulmonary lavage *in vivo* after inhalation of insoluble radioactive particles. In: *Inhaled Particles*, Part. IV, (Ed. Walton). Oxford: Pergamon Press, p. 597.

PETO, R. (1974). Guidelines on the analysis of tumour rates and death rates in experimental animals, *Br. J. Cancer*, **29**, 101.

PETO, R. (1977). Epidemiology, multistage models, and short term mutagenicity tests. In: *Origin of Human Cancer*, (Eds. Hiatt *et al.*). N.Y.: CSH Press, Cold Spring Harbor, p. 1403.

PETO, R. & others. (1980). Guidelines for simple sensitive significance tests for carcinogenic effects in long-term animal experiments. In: *Long-term and Short Term Screening Assays for Carcinogens: A critical Appraisal*, IARC Monographs, Suppl. 2, Lyon, p. 311.

POUR, P., STANTON, M.F., KUSCHNER, M., LASKIN, S. & SHABAD, L.M. (1976). Tumours of the respiratory tract. In: *Pathology of Tumours in Laboratory Animals*. Vol. I: Tumours of the Rat. Part 2., (Ed. Turusov). (IARC Scientific Publication no. 6), Lyon.

PREUSSMANN, R. (1976). Chemical carcinogens in the human environment, problems and quantitative aspects, *Oncology*, **33**, 51.

SAFFIOTTI, U., MONTESANO, R., SELLAKUMAR, A.R. & KAUKMAN, D.G. (1972a). Respiratory tract carcinogenesis induced in hamster by different dose levels of benzo(a)pyrene and ferric oxide, *J. Natl. Cancer Inst.*, **49**, 1199.

SAFFIOTTI, U., MONTESANO, R., SELLAKUMAR, A.R., CEFIS, F. & KAUKMAN, D.G. (1972b). Respiratory tract carcinogenesis in hamsters induced by different numbers of administrations of benzo(a)pyrene and ferric oxide. *Cancer Res.*, **32**, 1073.

SANDERS, C.L. (1973). Cocarcinogenesis of $^{239}PuO_2$ with chrysotile asbestos or benzopyrene in the rat abdominal cavity. In: *Radionuclide Carcinogenesis*. AEC Symposium series, (Eds. Sanders *et al.*) 138, CONF 720505, NTIS, USA: Springfield, Va.

SANDERS, C.L., DAGLE, G.E., CANNON, W.C., CRAIG, D.K., POWERS, G.J. & MEIER, D.M. (1976). Inhalation carcinogenesis of high-fired $^{239}PuO_2$ in rats, *Radiat. Res.*, **68**, 349.

SANDERS, C.L. & MAHAFFAY, J.A. (1979). Carcinogenicity of inhaled air-oxidized $^{239}PuO_2$ in rats, *Int. J. Radiat. Biol.*, **35**, 95.

SARACCI, R. (1977). Asbestos and lung cancer: an analysis of the epidemiological evidence on the asbestos-smoking interaction. *Int. J. Cancer*, **20**, 323.

SARACCI, R., (1981). Discussion of paper by Wahrendorf. In: *Perspective in Medical Statistics*, (Eds. Bithell & Coppi). London: Academic Press, p. 15.

TEMPLE, L.A., MARKS, S. & BAIR, W.J. (1960). Tumours in mice after pulmonary deposition of radioactive particles, *Int. J. Radiat. Biol.*, **2**, 143.

WHITTEMORE, A. & McMILLAN, A. (1983). Lung cancer mortality among US uranium miners: A reappraisal, *J. Natl. Cancer Inst.*, **71**, 489.